# The contrasted evolutionary fates of deep-sea chemosynthetic mussels (Bivalvia, Bathymodiolinae)^a^

**DOI:** 10.1002/ece3.749

**Published:** 2013-10-31

**Authors:** Justine Thubaut, Nicolas Puillandre, Baptiste Faure, Corinne Cruaud, Sarah Samadi

**Affiliations:** 1 Département Systématique et Evolution, Muséum National d'Histoire NaturelleUnité Mixte de Recherche 7138 (UPMC-IRD-MNHN-CNRS), “Systématique Adaptation et Evolution”, 75005, Paris, France; 2 Station Biologique de Roscoff, Unité Mixte de Recherche 7127, Centre National de la Recherche Scientifique, Université Pierre et Marie Curie29680, Roscoff, France; 3 BiotopeService Recherche et Développement, BP58 34140, Mèze, France; 4 Genoscope, CP 570691057 Evry, France

**Keywords:** Bathymodiolinae, chemosynthetic ecosystem, deep-sea, evolution

## Abstract

Bathymodiolinae are giant mussels that were discovered at hydrothermal vents and harboring chemosynthetic symbionts. Due to their close phylogenetic relationship with seep species and tiny mussels from organic substrates, it was hypothesized that they gradually evolved from shallow to deeper environments, and specialized in decaying organic remains, then in seeps, and finally colonized deep-sea vents. Here, we present a multigene phylogeny that reveals that most of the genera are polyphyletic and/or paraphyletic. The robustness of the phylogeny allows us to revise the genus-level classification. Organic remains are robustly supported as the ancestral habitat for Bathymodiolinae. However, rather than a single step toward colonization of vents and seeps, recurrent habitat shifts from organic substrates to vents and seeps occurred during evolution, and never the reverse. This new phylogenetic framework challenges the gradualist scenarios “from shallow to deep.” Mussels from organic remains tolerate a large range of ecological conditions and display a spectacular species diversity contrary to vent mussels, although such habitats are yet underexplored compared to vents and seeps. Overall, our data suggest that for deep-sea mussels, the high specialization to vent habitats provides ecological success in this harsh habitat but also brings the lineage to a kind of evolutionary dead end.

## Introduction

The exploration of the deep sea is relatively recent and many gaps remain in basic knowledge of marine biodiversity regarding taxonomy, geographic distribution, or ecology (Costello et al. 2010). Moreover, marine biologists are focused on a few emblematic environments such as Antarctic biota or hydrothermal vents which are characterized by peculiar physical and chemical conditions that represent physiological challenges for organisms. The consequence of such knowledge gaps is that uncommon and spectacular phenotypic features or behaviors are easily interpreted, under the Panglossian paradigm (Gould and Lewontin 1979), as adaptations to these peculiar environmental conditions. For example, it was speculated that the gigantism of pycnogonids from Antarctica is adaptive and stems from high polar oxygen availability coupled with low metabolic rates (Chapelle and Peck 1999), but physiological studies have not confirmed this hypothesis (Woods et al. 2009), which remains controversial (Klok 2009). An alternative explanation might be that it results from phylogenetic contingency rather than from adaptive processes. However, to disentangle adaptive evolution from phylogenetic inertia the diversity of the related organisms should be covered taxonomically, geographically, and ecologically (Van Buskirk 2009). For example, when sampling is biased toward a given habitat, characters shared by the taxa from this habitat may be mistakenly interpreted as an adaptation to it. Thus, because taxon sampling strongly affects the evolutionary inferences drawn from phylogenetic trees (Heath et al. 2008), closely related taxa, from others habitats and/or other regions, should be included in the phylogenetic analyses.

We here focus on the Bathymodiolinae mussels, often considered as model organisms in the study of adaptation to extreme marine environments (Kádár et al. 2005; Kadar and Powell 2006; Lallier 2006). Bathymodiolinae were first described from deep-sea hydrothermal vents that, contrasting with the more generally oligotrophic deep-sea habitats, are characterized by extreme physicochemical conditions associated with an abundant and unique fauna. Soon after the discovery of this environment, the impressive productivity was explained by the chemosynthetic process that sustains primary production in absence of light both through free-living bacteria, grazed by animals, and through bacteria living in symbiosis with metazoans (Jannasch 1985). Consequently, among the biological features of *Bathymodiolus* (the first genus described in the Bathymodiolinae to which are attributed most of the giant mussels sampled at hydrothermal vents), the symbiosis with chemosynthetic bacteria is often considered as the key adaptation that explains the ecological success of these mussels in these harsh habitats. This success was first explained (Craddock et al. 1995) as resulting from a gradual evolution toward specialization from shallow water environments to cold seeps and finally to deep-sea vents. Then, the evolutionary scenario was refined (Distel et al. 2000), based on a molecular phylogenetic analysis that included some small mussels sampled on organic remains, sunken at the deep-sea floor. This new scenario introduced a “wooden step” that predates the colonization of deep-sea seeps and vents. Recently, several studies (Duperron et al. 2009) revealed the presence of sulfide-oxidizing bacteria in the gills of all examined deep-sea mussels sampled on organic remains. Several phylogenetic studies also confirmed that the evolutionary history of vent and seep mussels is tightly tied to that of the small mussels sampled on organic falls of the deep sea and that the small mussels associated with organic falls should be included within Bathymodiolinae (Samadi et al. 2007; Lorion et al. 2009, 2010). These results, although still preliminary, suggest a more complex evolutionary history than gradual evolution (i.e., from shallow to deep or from standard oceanic conditions to “extreme” physical and chemical conditions). However, many studies (Jones et al. 2006; Kyuno et al. 2009; Fujiwara et al. 2010; Miyazaki et al. 2010) still suggest that gradual evolutionary scenarios in which, for example, the presence of intracellular chemosynthetic bacteria is interpreted as a “final step” toward the adaptation to extreme environments (c.f. Miyazaki et al. 2010).

Presently, most of the studies on Bathymodiolinae, focusing either on biology, physiology, or behavior, offer adaptive interpretations of biological features that do not take the most recent phylogenetic results into account (Dixon et al. 2004; Hardivillier et al. 2004; Pruski and Dixon 2007; Serafim et al. 2008; Mestre et al. 2009; Bettencourt et al. 2010). For example, to unravel genes specifically involved in hydrostatic pressure and chemosynthetic environmental adaptations, Bettencourt et al. (2010) compared the transcriptome profiles of *Bathymodiolus azoricus* and *Mytilus galloprovincialis*. The underlying assumption justifying the adaptive inferences is that these two species are “closely related Mytilid family members living in very distinct marine habitat.” However, a recent study (Lorion et al. 2010) confirmed that many biological features – such as the presence of chemosynthetic bacteria – are shared by more closely related mussels that do not inhabit the harsh vent habitat. Thus, adaptive inferences must be based on comparative approaches with these closest relatives rather than with the poorly related shallow water *Mytilus* species.

The main goal of this study was thus to clarify the phylogenetic relationships among the Bathymodiolinae *sensus lato* (i.e., including both Bathymodiolinae *sensus stricto* and the small mussels from organic falls included in the same monophyletic clade) from deep-sea environments and to propose a new classification at the genus level that reflects the evolutionary history. A barcoding approach was also used, including all species/Evolutionary Significant Units (ESUs) of Bathymodiolinae *sensu lato* available in the literature, in order to attempt to reassess a global classification at the genus level. This new classification aims at clarifying the context in which comparative studies of deep-sea mussels should be conducted to thoroughly test evolutionary hypotheses and thus to interpret biological features as adaptations. The phylogenetic analysis includes, in addition to the mitochondrial marker cytochrome oxydase I (COI) and the nuclear marker 28S rRNA used in the analysis performed by Lorion et al. (2010), one mitochondrial marker (16S rRNA) and four nuclear markers (18S rDNA, H3, HSP70, and Ant), for 51 terminal taxa belonging to Bathymodiolinae *s.l*. We extended the definition of Bathymodiolinae to include all deep-sea mytilids that are associated with chemosynthetic bacteria, but are not restricted to vents and seeps. The more finely resolved and strongly supported phylogeny of the Bathymodiolinae allowed us to explore the paths followed by these mussels during their evolution.

## Materials and Methods

### Taxon sampling

Taxon sampling is mainly based on Lorion et al. (2010) to which five additional terminal taxa were added. First, one specimen of the small mussels collected at the Juan de Fuca hydrothermal vents by McKiness et al. (2005) was included in the data set. These mussels were first attributed to the genus *Bathymodiolus* and labeled *B*. sp JdF (McKiness et al. 2005) and then to *Adipicola* (*Adipicola* MV in [Southward 2008; ] and *Adipicola* sp. JdF [Fontanez and Cavanaugh 2013; ]). Tissue samples of these mussels were provided by E. C. Southward. They are here labeled as *Benthomodiolus* sp. Juan de Fuca following the taxonomic revision proposed in J. Thubaut, R. von Cosel, S. Samadi, and P. Bouchet (unpubl. ms.) based on the phylogenetic results of this study. Second, two specimens collected off Papua New Guinea during the BioPapua cruise (Pante et al. 2012) and corresponding to two distinct morphospecies were added to the data set. These two specimens were collected off the Sepik River in Broken Bay together with many empty shells attributed to the two same morphospecies but also with other organisms typically found at cold seep sites (e.g., tubes of *Lamelibrachia sp*. and *Escarpia sp*., vesycomyid clams, and thyasirids). Both these two morphospecies were attributed to the genus *Gigantidas* (R. von Cosel, pers. comm.) and here referred to as *G*. sp.1 Broken Bay and *G*. sp.2 Broken Bay. We also have obtained four specimens assigned to the species *Idas simpsoni* from the collection of the Museum of London. These were trawled at 162 m in the northern Atlantic Ocean off Rockall between Iceland and Ireland, associated with whale bones. Last, an additional unstudied morphospecies was found within the collection of the MNHN. These mussels were registered in the MNHN database as collected on vertebrate bones in South Atlantic at 3900 m depth. The shells are similar to those of *Benthomodiolus* mussels (R. von Cosel, pers. comm.). Following Samadi et al. (2007), *Modiolus modiolus* was used as an outgroup. All specimens corresponding to the 51 terminal taxa used in this study are listed in the Table 1.

**Table 1:** Specimen collection sites, maximum shell size, and GenBank accession numbers (sequences obtained in this study are indicated with stars) for Mytilidae included in phylogenetic analyses

Genus	Species	Ocean	Depth range (m)	Habitat	Maximum shell size (mm)	COI	28S	18S	16S	H3	HSP70	Ant
*Modiolus*	*modiolus*	Atlantic	10–300	Intertidal subtidal	**147**	FJ890501^(1)^	EF526455^(2)^	KF611701*	KF611732*	KF720595*		KF720516*
*Benthomodiolus*	*lignocola*	Western Pacific	1180	Wood, bone	18.1	AY275545^(3)^	AY781131^(4)^	AF221648^(5)^	KF611733*	KF720596*		
	sp. Juan de Fuca	Eastern Pacific	2420	Vent	16	KF611694*	KF611699*	KF611702*	KF611734*	KF720597*		
	sp. South Atlantic	Atlantic	3900	Bone	8.8	KF611691*	KF611698*	KF611703*	KF611733*	KF720593*	KF720560*	
*Vulcanidas*	ESU E	Western Pacific	150–785	Wood	36.7	FJ937079^(1)^	GU065791^(1)^	KF611704*	KF611736*	KF720598*	KF720592*	KF720517*
	ESU F	Western Pacific	275–560	Wood	35	FJ937127^(1)^	GU065809^(1)^	KF611705*	KF611737*	KF720599*	KF720578*	KF720518*
	SAL3	Western Pacific	400–1085	Wood	38.3	DQ340772^(6)^	DQ863946^(1)^	DQ340800^(6)^	KF611738*	KF720600*	KF720582*	KF720519*
	*insolatus*	Western Pacific	140–504	Vent	**178**	FJ767936^(1)^	FJ767937^(1)^	KF611706*	KF611739*	KF720601*	KF720558*	KF720520*
*Tamu*	*fisheri*	Atlantic	546–650	Seep	**58**	AY649803^(4)^	AY781132^(4)^	AF221642^(5)^				
*Lignomodiolus*	ESU S'	Western Pacific	230–380	Wood	18.8	FJ937240^(1)^	GU065816^(1)^					
	ESU S''	Western Pacific	190–400	Wood	18.8	FJ937258^(1)^	GU065829^(1)^	KF611707*	KF611740*	KF720602*	KF720590*	KF720521*
	SAL4	Western Pacific	94–886	Wood	25	DQ340776^(6)^	DQ863947^(6)^	DQ340796^(6)^	KF611741*	KF720603*	KF720589*	KF720522*
	ESU R	Western Pacific	490	Wood	8.2	FJ937239^(1)^	GU065877^(1)^	KF611708*	KF611742*	KF720604*	KF720588*	KF720523*
	ESU Q	Western Pacific	100–130	Wood	7.9	FJ937230^(1)^	GU065875^(1)^	KF611709*	KF611743*	KF720605*	KF720561*	KF720524*
	ESU G	Western Pacific	150–670	Wood	9.6	FJ937161^(1)^	GU065778^(1)^	KF611710*	KF611744*	KF720606*	KF720591*	KF720525*
*Gigantidas*	sp.1 Broken Bay	Western Pacific	361–750	Seep	**85**	KF611693*	KF611696*		KF611745*	KF720607*	KF720555*	KF720526*
	*taiwanensis*	Western Pacific	200–355	Vent	**56**	GU966638^(1)^	GU966641^(1)^	KF611711*	KF611746*	KF720608*	KF720566*	KF720527*
	*mauritanicus*	Atlantic	540–2222	Seep	**127**	FJ890502^(1)^	FJ890504^(1)^	KF611712*	KF611747*	KF720609*	KF720565*	KF720528*
	*tangaroa*	Western Pacific	920–1205	Seep	**200**	AY608439^(3)^	AY781149^(4)^	AY649820^(4)^	KF611748*	KF720610*	KF720572*	KF720529*
	sp.2 Broken Bay	Western Pacific	361–750	Seep	**75.7**	KF611692*	KF611697*	KF611713*	KF611749*	KF720611*	KF720562*	KF720530*
	*gladius*	Western Pacific	300–460	Vent	**316**	AY649802^(4)^	AY781134^(4)^	AY649821^(4)^				
	*crypta* (B')	Western Pacific	441	Wood, bone	30	EU702319^(2)^	EU683298^(2)^	KF611714*	KF611750*	KF720612*	KF720563*	KF720531*
	*crypta* (B'')	Western Pacific	431–493	Wood, bone	30	EU702315^(2)^	EU683301^(2)^		KF611751*	KF720613*		KF720532*
*Nypamodiolus*	*longissimus*	Western Pacific	400–1767	Wood	42	DQ340773^(6)^	DQ863945^(6)^	DQ340798^(6)^	KF611752*	KF720614*	KF720564*	KF720533*
	ESU J	Western Pacific	360–370	Wood	10	FJ937189^(1)^	GU065842^(1)^	KF611715*	KF611753*	KF720615*	KF720577*	KF720534*
	ESU I	Western Pacific	190–567	Wood	8.6	FJ937188^(1)^	GU065774^(1)^	KF611716*	KF611754*	KF720616*	KF720576*	KF720535*
	ESU H	Western Pacific	220–560	Wood	10	FJ937073^(1)^	GU065856^(1)^	KF611717*	KF611755*	KF720617*	KF720575*	KF720536*
	*simpsoni*	Atlantic	162	Bone	23	KF611695*	KF611700*	KF611731*	KF611773*	KF720594*	KF720571*	KF720554*
*“Bathymodiolus”*	*manusensis*	Western Pacific	1627	Vent	**86.2**	GU966637^(1)^	GU966642^(1)^	KF611718*		KF720618*	KF720556*	KF720537*
*Terua*	*arcuatilis*	Western Pacific	880	Bone	29	FJ937033^(1)^	GU065879^(1)^	KF611719*	KF611756*	KF720619*	KF720584*	KF720538*
	ESU T	Western Pacific	800–1060	Bone	6.2	FJ937283^(1)^	GU065804^(1)^	KF611720*	KF611757*	KF720620*	KF720585*	KF720539*
*Bathymodiolus*	*puteoserpentis*	Atlantic	3023–3510	Vent	**119**	AY649796^(4)^	AY781151^(4)^	AF221640^(5)^				
	*azoricus*	Atlantic	866–2330	Vent	**119**	AY649795^(4)^	AY781148^(4)^	AY649822^(4)^	KF611758*	KF720621*	KF720580*	KF720540*
	*heckerae*	Atlantic	3314	Seep	**230**	AY649794^(4)^	AY781138^(4)^	AF221639^(5)^				
	*boomerang*	Atlantic	1000–3170	Seep	**360**	FJ890503^(1)^	FJ890505^(1)^		KF611759*	KF720622*	KF720579*	KF720541*
	*brevior*	Western Pacific	3589	Vent	**140**	AY649799^(4)^	AY781150^(4)^	AY649824^(4)^				
	*thermophilus*	Eastern Pacific	2460–2747	Vent	**200**	GU966639^(1)^	GU966640^(1)^		KF611760*	KF720623*		FJ842134^(7)^
	aff. *thermophilus*	Eastern Pacific	2331	Vent		AF456317^(8)^	AY781140^(4)^					
	*brooksi*	Atlantic	2222–3314	Seep	**180**	AY649798^(4)^	AY781135^(4)^					
*Idas*	SAL1	Western Pacific	408–1356	Wood, bone	8	DQ340775^(6)^	DQ863944^(6)^	DQ340794^(6)^	KF611761*	KF720624*	KF720567*	KF720542*
	ESU P	Western Pacific	180–1390	Wood	8.7	FJ937222^(1)^	GU065846^(1)^	KF611721*	KF611762*	KF720625*	KF720557*	KF720543*
	ESU O	Western Pacific	473–890	Wood	6.7	FJ937211^(1)^	GU065763^(1)^	KF611722*	KF611763*	KF720626*	KF720581*	KF720544*
	ESU K	Western Pacific	500–540	Wood	6	FJ937192^(1)^	GU065868^(1)^	KF611723*	KF611764*	KF720627*	KF720568*	KF720545*
	*washingtonius*	Eastern Pacific	960–1910	Vent, wood, bone	8.6	AY275546^(3)^	AY781146^(4)^	AF221645^(5)^				
	sp. D	Western Pacific	556–1724	Wood	15.6	EU702357^(2)^	EU683275^(2)^	KF611724*	KF611765*	KF720628*	KF720587*	KF720546*
	sp. C	Western Pacific	275–1285	Wood, bone	16.7	EU702376^(2)^	EU683260^(6)^	KF611725*	KF611766*	KF720629*	KF720586*	KF720547*
	ESU M	Western Pacific	590–720	Wood	10.8	FJ937202^(1)^	GU065845^(1)^	KF611726*	KF611767*	KF720630*	KF720583*	KF720548*
	ESU L	Western Pacific	450–1010	Wood	8.6	FJ937193^(1)^	GU065767^(1)^	KF611727*	KF611768*	KF720631*	KF720574*	KF720549*
	ESU N	Western Pacific	800–1290	Wood, bone	10	FJ937205^(1)^	GU065843^(1)^	KF611728*	KF611769*	KF720632*	KF720573*	KF720552*
	*iwaotakii (*A')	Western Pacific	441–1866	Wood, bone	10	EU702333^(2)^	EU683288^(2)^	KF611729*	KF611770*	KF720633*	KF720559*	KF720550*
	*iwaotakii* (A'')	Western Pacific	490–2307	Wood	10	EU702322^(2)^	EU683295^(2)^		KF611771*	KF720634*	KF720569*	KF720551*
	*macdonaldi*	Atlantic	650	Seep	6.7	AY649804^(4)^	AY781145^(4)^	AF221647^(5)^				
	*modiolaeformis*	Mediterranean	2129	Seep	13	FJ158585^(9)^	FJ159555^(1)^	KF611730*	KF611772*	KF720635*	KF720570*	KF720553*

Shell sizes higher than 50 mm are given in bold. References for sequences: (1) Lorion et al. (2010), (2) Lorion et al. (2009), (3) Smith et al. (2004), (4) Jones et al. (2006), (5) Distel et al. (2000), (6) Samadi et al. (2007), (7) Audzijonyte and Vrijenhoek (2009), (8) Won et al. (2003) and (9) Lorion et al. (2012). COI, cytochrome oxydase I.

### Molecular methods

For the five specimens not included in the study of Lorion et al. (2010), DNA was extracted from whole specimens (or gills only for the largest specimens) using the QIAmp® DNA Micro Kit (Qiagen, Valencia, CA). For these specimens, the two gene fragments used in Lorion et al. (2010) were amplified: (1) the COI mitochondrial gene using the universal primer LCO1490 (Folmer et al. 1994) and the reverse primer H691 (5′-GTRTTAAARTGRCGATCAAAAAT-3′) designed for deep-sea mussels (Duperron et al. 2008a) and (2) a fragment of the 28S rRNA nuclear gene, covering the D1, D2, and D3 domains (Hassouna et al. 1984) using primers C1′ (5′-ACCCGCTGAATTTAAGCAT-3′) and C4 (5′-TCGGAGGGAACCAGCTACTA-3′). For the whole set of specimens, five additional gene fragments were analyzed: (1) a fragment of the 16S rRNA mitochondrial gene was amplified using the universal 16S primer LRJ-12864 (5′-CTCCGGTTTGAACTCAGATCA-3′) and the primer Idas 16SA (5′-GGARGTASGCCCTGCCCWATGC-3′) designed by Baco-Taylor (2002). Although many data are available for the ND4 mitochondrial gene, we did not use this gene that has been shown to be highly saturated (Samadi et al. 2007). (2) The 18S rDNA nuclear gene was amplified in three overlapping fragments using three pairs of primers: 1F and 5R, 3F and Bi, A2 and 9R (Giribet et al. 1996; Distel 2000; Okusu et al. 2003), (3) a fragment of the histone nuclear gene H3 was amplified using the primers H3F1 (5′-ATGGCTCGTACCAAGCAGACVGC-3′) and H3R1 (5′-ATATCCTTRGGCATRATRGTGAC-3′), (4) a fragment of the 70-kDa heat-shock protein (HSP70) nuclear gene was amplified using primers HSP70F (5′-GGGAAAGTTGACATTATTGCCAATG-3′) and HSP70R (5′-ATTCATAAATTCTGTCAACATTTTCTGT-3′) (developed by BF), and (5) a fragment of the gene encoding the adenosine nucleotide (ADP/ATP) translocase (Ant) was amplified using the pair of primers designed by Audzijonyte and Vrijenhoek (2009). Polymerase chain reaction (PCR) were performed in a 25 μL, final volume, containing approximately 3-ng template DNA, 1.5-mmol/L MgCl_2_, 0.26 mmol/L of each dNTPs, 0.3 μmol/L of each primer, 5% dimethyl sulfoxide (DMSO), and 0.75 unit of Taq Polymerase (Qbiogene, Illkirch, France) Amplification products were generated by an initial denaturation step of 4 min at 94°C followed by 30–35 cycles of denaturation at 94°C for 40 sec, annealing at 48°C for ND4 gene, 50°C for COI and first and second fragment of 18S gene, 52°C for 28S gene and third fragment of 18S gene, 55°C for 16S, H3 and HSP70 genes for 50 sec, and extension at 72°C for 1 min. A final elongation was performed at 72°C for 10 min. The gene Ant was amplified according to PCR conditions described in Audzijonyte and Vrijenhoek (2009). All PCR products were purified and sequenced at the Genoscope for both DNA strands. The 203 new sequences were deposited in BOLD and GenBank.

### Phylogenetic analyses

Chromatograms were edited with Sequencher 4.1.4, DNA sequences were aligned with the Clustal W module in Mega 4.0 (Tamura et al. 2007), and ambiguous sites were excluded. For each of the seven genes, one specimen of each species or ESUs (Samadi et al. 2007; Lorion et al. 2009, 2010) was used to evaluate the phylogenetic relationships among them. Best-fitting substitution models were estimated for each gene separately and for a data set that combined the seven single-gene data sets using MrModelTest 3.7 with the Akaike information criterion (AIC) (Posada and Crandall 1998). Each of the seven single-gene data sets and the combined data set were fitted to a general time-reversible model with a proportion of invariant sites and gamma-distributed rates (GTR+I+Γ). Phylogenetic reconstructions were performed, for both single-gene data sets and the combined data set, using maximum likelihood (ML) and Bayesian Inference (BI) approaches. ML analyses were performed using the software Treefinder (Jobb et al. 2004) and robustness of the nodes was assessed using nonparametric bootstrapping (1000 replicates) (Felsenstein 1985). BI analyses were performed using MrBayes (Ronquist and Huelsenbeck 2003) as implemented on the Bioportal cluster (University of Oslo, Bioportal: http://www.bioportal.uio.no). Eight Markov chains and two parallel analyses were run over 50M generations, sampled every 5000th step, and associated with a heating temperature of 0.01. To assess if the two independent runs converged, likelihood curves and standard deviation of split frequencies were analyzed using Tracer v1.4.1 (Rambaud and Drummond 2007) and AWTY website (http://king2.scs.fsu.edu/CEBProjects/awty/awty_start.php) (Nylander et al. 2008).

### Molecular assignation of species to genera

The taxon sampling used for the phylogenetic analysis covers the lineage diversity but not for all lineages the species diversity. Thus, to compare the species diversity among redefined genera we use a DNA barcoding–like approach (i.e., based on COI data), to assign species to genera. We first gathered from World Register of Marine Species (WORMS; http://www.marinespecies.org) a provisional list of valid names for each genus, and gathered, when available, the associated COI data from GenBank. Second, we gathered the COI data for the species identified in the literature using molecular data, but that have not yet been attributed to species names. This set of species was identified by Miyazaki et al. (2004), Iwasaki et al. (2006), Fujita et al. (2009), and Kyuno et al. (2009) using the COI and ND4 gene fragments. These taxa were labeled according to Kyuno et al. (2009) and were, first, tentatively attributed to either named species or ESUs of our data set. In the literature, the mean intraspecific genetic distance estimated using the Kimura 2-parameters (K2P) distance was about ∼1% in Won et al. (2003) and Miyazaki et al. (2004), and of 1.8% in Lorion et al. (2010). We thus used a conservative threshold of 2% K2P genetic distance to attribute a sequence to defined species or ESU. Second, an extended COI data set was constituted to perform a phylogenetic analysis. This data set included a reference data set (i.e., one sequence per named species or ESUs of our data set that are all attributed to a genus name based on the seven-genes phylogeny), and all COI data gathered from GenBank. This extended COI data set was analyzed using BI and ML approaches (see Table 2) to determine among the reference data set the closest relatives of each of the additional species or ESUs gathered from the literature. The attribution to a genus name was based on the sister relationship with a species – or a clade of species – of the reference data set. For one species name (*B. elongatus*), no COI data were available, although data for another gene fragment (ITS2) were available in GenBank. In this case, the attribution to genus followed the result of the phylogenetic analysis provided by the authors (see Table 2). Last, for named species for which no genetic data were available, species were tentatively placed in genera considering the available morphological data.

**Table 2:** Specimen collection sites, GenBank accession numbers for Bathymodiolinae included in the Barcode analysis (sequences obtained in this study are indicated with stars), and localization of symbionts

Genus	Species	Locality	Habitat	Depth (m)	COI	ITS2	Localization of symbionts
*Benthomodiolus* Dell, 1987	lignocola* Dell, 1987	WP	w, b	**1180**	AY275545^(1)^		
	*geikotsucola* Okutani & Miyazaki, 2007	WP	b	**4051**	AB257513^(2)^		
	*abyssicola* (Knudsen, 1970)	EP	w	**3270**–**3670**			
	**sp. Juan de Fuca**	EP	v	**2420**	KF611694^(3)^		Extracellular?^(12)^
	**sp. South Atlantic**	SA	b	**3900**	KF611691^(3)^		
*Vulcanidas* Cosel and Marshall, 2010	insolatus* Cosel and Marshall, 2010	WP	v	140–504	FJ767936^(4)^		Intracellular?^(13)^
	**SAL3**	WP	w	400–1085	DQ340772^(5)^		
	**ESU E**	WP	w	150–785	FJ937079^(4)^		
	**ESU F**	WP	w	275–560	FJ937127^(4)^		
*Tamu* Gustafson, Turner, Lutz & Vrijenhoek, 1998	fisheri* Gustafson, Turner, Lutz & Vrijenhoek, 1998	GM	s	546–650	AY649803^(6)^		
*Lignomodiolus* Cosel & Thubaut in prep.	miniboomerang* **(ESU G)** Cosel & Thubaut in prep.	WP	w	150–670	FJ937161^(4)^		Extracellular^(14)^
	**ESU S'**	WP	w	230–380	FJ937240^(4)^		
	**ESU S''**	WP	w	190–400	FJ937245^(4)^		
	**SAL4**	WP	w	94–886	DQ340776^(5)^		
	**ESU R**	WP	w	490	FJ937238^(4)^		
	**ESU Q**	WP	w	100–130	FJ937229^(4)^		
*Gigantidas* Cosel & Marshall, 2003	gladius* Cosel & Marshall, 2003	WP	v	300–460	AY649802^(6)^		Intracellular^(15)^
	*horikoshii* Hashimoto & Yamane, 2005	WP	v	486	AB257538^(2)^		
	Nikko *G*. sp.	WP	v	485	AB257544^(2)^		
	Sumisu *G*. sp.	WP	v	676–686	AB257553^(2)^		
	Ashizuri sp.	WP	s	575	AB257529^(2)^		
	*childressi* Gustafson, Turner, Lutz & Vrijenhoek, 1998	GM	s	**1859**	AB257532^(2)^		Intracellular^(16)^
	*platifrons* Hashimoto & Okutani, 1994	WP	v, s	**1180**	AB101419^(7)^		Intracellular^(17)^
	*japonicus* Hashimoto & Okutani, 1994	WP	s	**1170**	AB101422^(7)^		Intracellular^(17)^
	***mauritanicus*** Cosel, 2002	NA	s	540–2222	FJ890502^(4)^		
	***tangaroa*** Cosel & Marshall, 2003	WP	s	920–1205	AY608439^(6)^		
	Sissano *B*. sp.3	WP	s	**1881**	AB257551^(2)^		
	*securiformis* Okutani, Fujikura & Sasaki, 2003	WP	s	637–642	AB170048^(8)^		
	*hirtus* Okutani, Fujikura & Sasaki, 2003	WP	s	637–642	AB170047^(8)^		
	*anteumbonatus* Cosel & Janssen, 2008	WP	s	**1574**–**1628**			
	*edisonensis* Cosel & Janssen, 2008	WP	s	**1574**–**1629**			
	***taiwanensis*** Cosel, 2008	WP	v	271	GU966638^(4)^		
	Kikaijima *B*. sp.	WP	s	**1430**	AB257556^(2)^		
	Sissano *B*. sp.1	WP	s	**1646**	AB257548^(2)^		
	Sissano *B*. sp.2	WP	s	**1881**	AB257547^(2)^		
	Chamorro *B*. sp.	WP	s	**2899**	AB257530^(2)^		
	***crypta*** **(B' and B'')** Dall, Bratsch & Rehder, 1938	WP	w, b	431–493	EU702315^(9)^		Intracellular^(9)^
	**sp.1 Broken Bay**	WP	s	361–750	KF611693^(3)^		
	**sp.2 Broken Bay**	WP	s	361–750	KF611692^(3)^		
	Aitape *G*. sp.	WP	s	470	AB257524^(2)^		
*Nypamodiolus* Cosel & Thubaut in prep	longissimus* (Thiele & Jaeckel, 1931)	WP	w	400–1767	DQ340783^(5)^		Intracellular^(14)^
	**ESU J**	WP	w	360–370	FJ937189^(4)^		
	**ESU I**	WP	w	190–567	FJ937182^(4)^		
	*japonica* Habe, 1967 **(ESU H)**	WP	w	150–110	FJ937060^(4)^		
	*simpsoni* (Marshall, 1900)	NA	b	162	KF611695^(3)^		
*“Bathymodiolus”*	***manusensis*** Hashimoto & Furuta 2007	WP	v	**1627**	GU966637^(4)^		
	Lau *B*. sp.	WP	v	**1818**	AB257539^(2)^		
	NZ *B*. sp.	WP	v	unknown	AB255739^(2)^		
	*aduloides* Hashimoto & Okutani 1994	WP	v	**1378**–**1451**	AB170054^(8)^		Intracellular^(18)^
*Terua* Dall, Bratsch & Rehder, 1938	pacifica* Dall, Bratsch & Rehder, 1938	WP	v	229	AB170040^(8)^		Extracellular^(19)^
	***arcuatilis*** Dell, 1995	WP	v	880	FJ937033^(4)^		
	**ESU T**	WP	v	800–1060	FJ937275^(4)^		
*Bathymodiolus* Kenk & Wilson, 1985	thermophilus* Kenk & Wilson, 1985	EP	v	**2460**–**2747**	GU966639^(4)^		Intracellular^(20)^
	***azoricus*** Cosel & Comtet, 1999	NA	v	866–2330	AY649795^(6)^		Intracellular^(21)^
	***puteoserpentis*** Cosel, Métivier & Hashimoto, 1994	NA	v	**3023**–**3510**	AY649796^(6)^		Intracellular^(22)^
	*elongatus* Cosel, Métivier & Hashimoto, 1994	WP	v	**2765**		DQ513471^(11)^	
	***brevior*** Cosel, Métivier & Hashimoto, 1994	WP	v	**3589**	AY649799^(6)^		Intracellular^(23)^
	*marisindicus* Hashimoto, 2001	I	v	**2454**	AB170042^(8)^		Intracellular^(24)^
	*septendierum* Hashimoto & Okutani, 1994	WP	v	**1288**	AB101424^(2)^		Intracellular^(17)^
	***boomerang*** Cosel and Olu 1998	NA	v	**1000**–**3170**	FJ890503^(4)^		Intracellular^(25)^
	***heckerae*** Gustafson, Turner, Lutz & Vrijenhoek, 1998	GM	v	**3314**	AY649794^(6)^		Intracellular^(26)^
	***brooksi*** Gustafson, Turner, Lutz & Vrijenhoek, 1998	GM	v	**2222**–**3314**	AY649798^(6)^		Intracellular^(26)^
*Idas* Jeffreys, 1876	argenteus* Jeffreys, 1876	NA	w	**1836**			
	***macdonaldi*** Gustafson, Turner, Lutz & Vrijenhoek, 1998	GM	s	650	AY649804^(6)^		Intracellular^(15)^
	***modiolaeformis*** (Sturany, 1896)	M	s	**2129**	EF210072^(10)^		Intracellular?^(27)^
	***iwaotakii*** **(A' and A'')** (Habe, 1958)	WP	w, b	441–2307	EU702322^(9)^ EU702323^(9)^		
	***washingtonius*** (Bernard, 1978)	EP	v, w and b	960–1910	AY275546^(1)^		Intracellular^(28)^
	**sp. SAL1**	WP	w, b	408–1356	FJ937271^(5)^		
	**ESU P**	WP	w	180–1390	FJ937213^(4)^		
	**ESU O**	WP	w	473–890	FJ937206^(4)^		
	**ESU K**	WP	w	500–540	FJ937191^(4)^		
	**sp. D**	WP	w	556–1724	EU702350^(9)^		Extracellular^(29)^
	**sp. C**	WP	w, b	275–1285	EU702360^(9)^		Extracellular^(9)^
	**ESU M**	WP	w	590–720	FJ937202^(4)^		
	**ESU L**	WP	w	450–1010	FJ937193^(4)^		
	**ESU N**	WP	w, b	800–1290	FJ937203^(4)^		

Species/ESUs used in the multigenic analyses are given in bold. Stars indicated the type species of the various genera. Depth scales higher than 1000 m are given in bold. Species underlined are labeled following (19). For locality: EP, Eastern Pacific; GM, Gulf of Mexico; I, Indian Ocean; M, M Mediterranean Sea; NA, Northern Atlantic; SA, Southern Atlantic; and WP, Western Pacific. For habitat: w, wood; b, bone; v, vent; and s, seep. Reference for host sequence and symbiont localization: (1) Smith et al. (2004), (2) Miyazaki et al. (2010), (3) this study, (4) Lorion et al. (2010), (5) Samadi et al. (2007), (6) Jones et al. (2006), (7) Miyazaki et al. (2004), (8) Iwasaki et al. (2006), (9) Lorion et al. (2009), (10) Duperron et al. (2008a), (11) Olu-Le Roy et al. (2007), (12) McKiness et al. (2005), (13) von Cosel and Marshall (2010), (14) Duperron et al. (2008b), (15) Won et al. (2008), (16) Childress et al. (1986), (17) Fujiwara et al. (2000), (18) Yamanaka et al. (2000), (19) Fujiwara et al. (2010), (20) Distel et al. (1988), (21) Fiala-Medioni et al. (2002), (22) Cavanaugh et al. (1992), (23) Dubilier et al. (1998), (24) Yamanaka et al. (2003), (25) Duperron et al. (2005), (26) Duperron et al. (2007), (27) Olu-Le Roy et al. (2004), (28) Deming et al. (1997), and (29) Duperron et al. (2009). COI, cytochrome oxydase I.

### Character evolution

Ancestral character state reconstruction of habitat use and depth was conducted using the ML method implemented in Mesquite 2.75 (Maddison and Maddison 2011). Taxa were assigned to four different habitats (hydrothermal vent, cold seep, organic falls, nonchemosynthetic environment). The character “habitat” was coded as “0″ for nonchemosynthetic environment, “1” for hydrothermal vent, “2” for cold seep, and “3” for organic remain. The character “depth” was coded according to Craddock et al. (1995) and Jones et al. (2006) in taking the shallowest recorded depth lesser (“0”) and greater than 1000 m (“1”). Ancestral states were reconstructed for all Bayesian trees retained from the analysis of the combined data set and their mean likelihood was then plotted on the maximum clade credibility tree.

## Results

### Phylogenetic relationships among mytilids associated with deep-sea reducing habitats and genus-level classification

**Figure 1:**
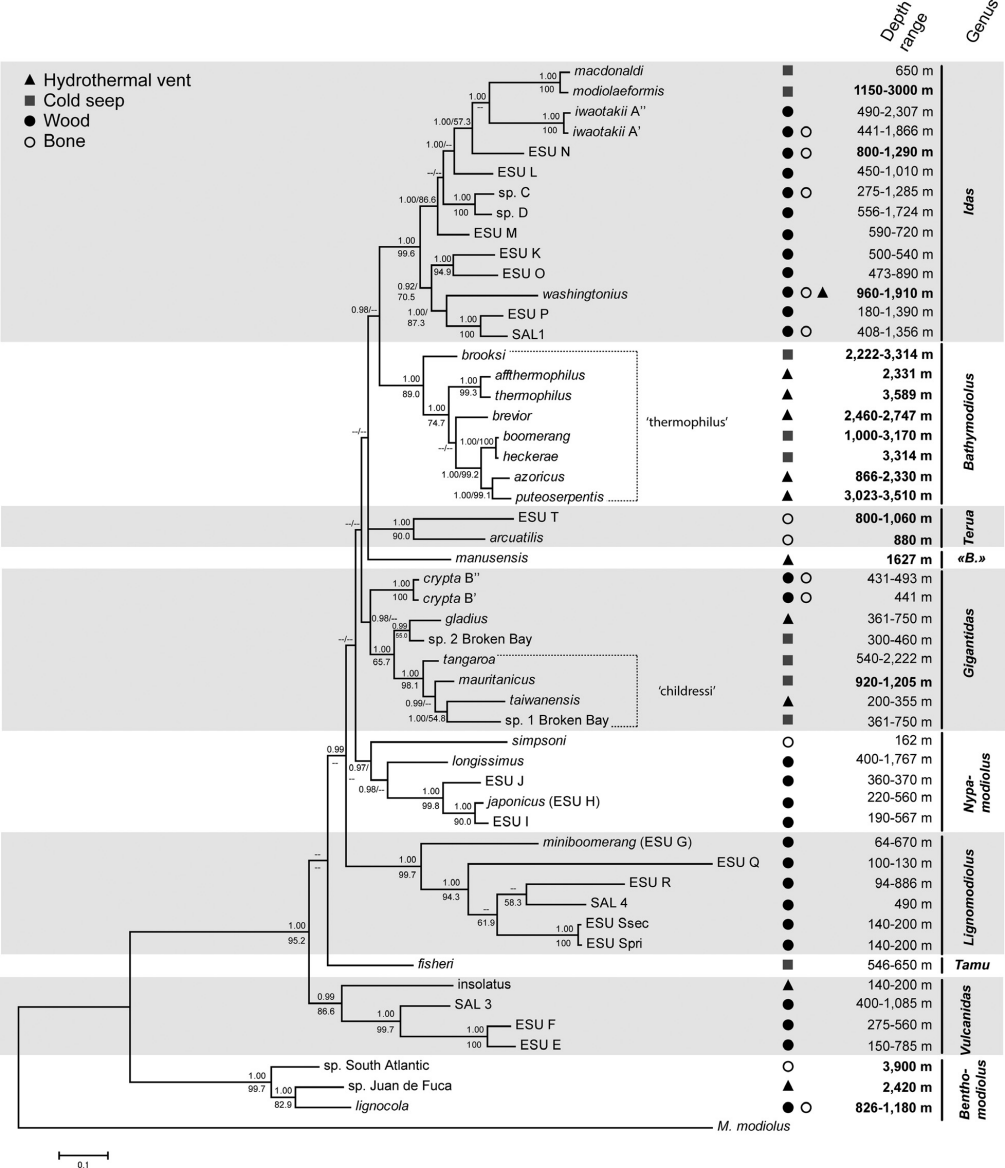
Phylogenetic relationships among Bathymodiolinae obtained from Bayesian analysis of the multigene data set (two mitochondrial and five nuclear genes), including species from hydrothermal vents, cold seeps, and organic falls available on GenBank and new samples. Posterior probabilities (PP) and bootstraps values obtained from maximum likelihood (ML) analysis are given above and below nodes, respectively. PP and bootstraps values lower than 0.90 and 50%, respectively, are not shown. Scale bar represents 0.1% estimated base substitution. Environments and depth range inhabited by each species are given. *B., Bathymodiolus*.

Maximum likelihood and BI of each of the individual gene fragments provided similar patterns of relationships, with no incongruence, but poorly resolved phylogenetic trees, whereas the multigene alignment of 51 taxa/ESUs and 5912 bp produced 10 robustly supported clades (Fig. 1).

A first monophyletic lineage (99.7% of bootstraps, posterior probabilities (PP) = 1) was sister group to all the other mussels from chemosynthetic habitats. This lineage included *Be. lignocola* but also two additional undescribed species (sp. Juan de Fuca and sp. South Atlantic). As *Be. lignocola* is the type species for *Benthomodiolus*, both sp. Juan de Fuca and sp. South Atlantic were attributed to this genus.

All the other lineages were included in a well-suported clade (95.2% of bootstraps, PP = 1). The position of type species was used to name six lineages: *Vulcanidas* Cosel & Marshall 2010*, Tamu* Gustafson, Turner, Lutz & Vrijenhoek 1998, *Lignomodiolus* Cosel & Thubaut in prep., *Nypamodiolus* Cosel & Thubaut in prep.*, Gigantidas* Cosel & Marshall 2003, and *Bathymodiolus* Kenk & Wilson 1985 (Fig. 1). The species *Terua pacifica*, type species of the genus *Terua*, clustered with the species *arcuatilis* and the ESU T based on the COI and ND4 genes (as revealed by the DNA barcoding approach – see below), and the name *Terua* was thus attributed to this clade. The sister clade of *Bathymodiolus* mainly included species generally attributed to *Idas*, and we applied this name to the clade. For only one lineage no genus name could be applied. The species *“B.” manusensis* is alone and we refrain to describe it as a new genus pending for additional species to be recognized as belonging to this lineage.

We also recovered the lineage, first, highlighted by Jones et al. (2006) and labeled as the “childressi” group: it fell here within the *Gigantidas* lineage. The two morphospecies collected off the Bismarck Sea in the Broken bay (Papua New Guinea) also clustered within the *Gigantidas* lineage: *G*. sp.1 Broken Bay was robustly included within the “childressi” group (Jones et al. 2006) as the sister species of *G. taiwanensis,* and *G*. sp.2 Broken Bay was the sister species of *G. gladius*. Finally, the specimens attributed to *Idas simpsoni* from the northern Atlantic clustered in the *Nypamodiolus* lineage and thus this species must be included in this genus.

### Species diversity within genera

**Figure 2:**
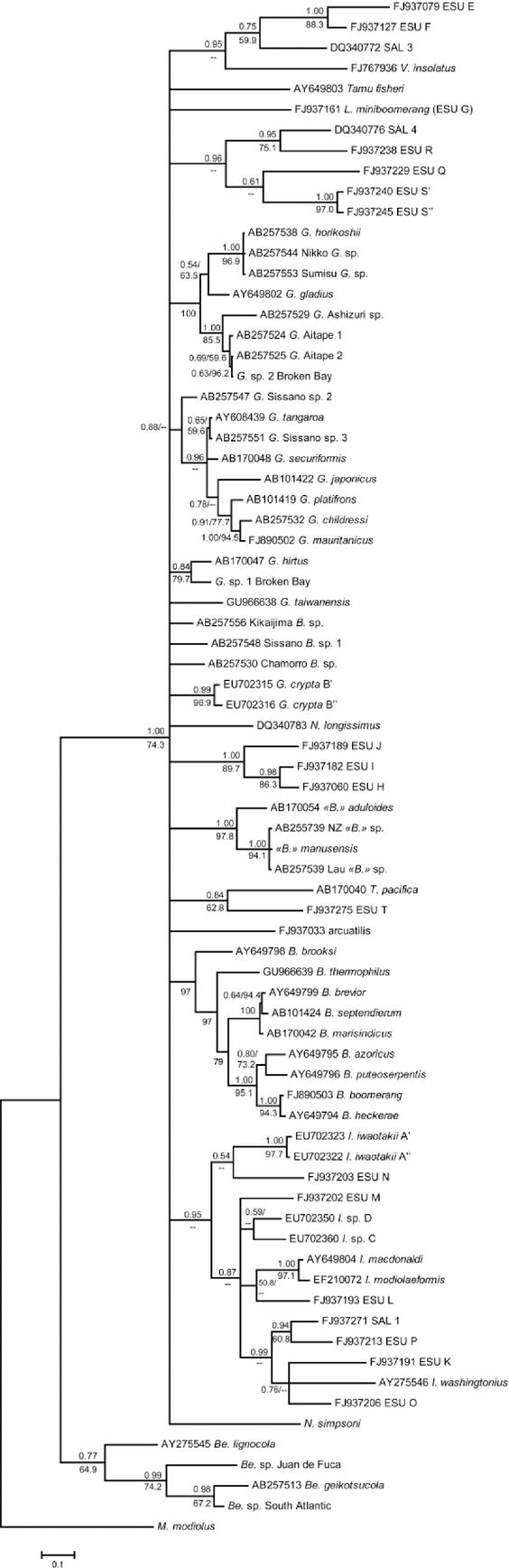
Bayesian tree obtained from the analysis of the Cytochrome Oxydase I (COI) mt DNA data set (see Table 2). Only PP (right) higher than 0.50 and bootsraps values (left) obtained from ML analysis higher than 50% are given. Scale bar represents 0.1% estimated base substitution.

Six species identified in the literature by molecular data were attributed to either a named species or an ESU of our data set using the 2% K2P threshold: NZ *B*. sp. and Lau *B*. sp. both fall with *“B.” manusensis,* Nikko *G*. sp. and Sumisu *G*. sp. both fall with *G. horikoshii*, Sissano *B*. sp. 3 falls with *G. tangaroa*, and finally Aitape *G*. sp. falls with *Gigantidas* sp.2 Broken Bay (see Table 2 and Fig. 2). Based on mitochondrial data and using the same threshold, Van Dover et al. (2001) suggested that *B. brevior* and *B. marisindicus* might be conspecific. More recent studies shown that these two species and *B. septemdierum* are closely related and are connected by gene flows (see Kyuno et al. 2009 and Miyazaki et al. 2010). Thus, considering all the data gathered for this three putative species, we agree with Vrijenhoek (2009) that they might be conspecific. The name *B. septemdierum* is the oldest name and has the nomenclatural priority over the two others. Similarly, *B. boomerang* and *B. heckerae* in one hand and *G. childressi* and *G. mauritanicus* in the other hand belong to two complexes of very closely related species that although geographically distant might not be reproductively isolated (Olu-Le Roy et al. 2007) and could thus be conspecific. A similar pattern of weak structure over large geographic has been detected for the small sunken wood mussels (Lorion et al. 2010), but contrary to these studies the pairs of populations geographically very distant but genetically weakly distant have been considered as unique ESUs.

The attribution based on the distance threshold was confirmed for most of the added species by the phylogenetic analysis of the extended COI data set (Fig. 2). The only three exceptions are Chamorro *B*. sp., Kikaijima *B*. sp., and Sissano *B*. sp. 1 for which the sister taxa could not be determined using the COI data set. These three taxa are, however, robustly placed in the “childressi” group by Miyazaki et al. (2010), using a combined phylogenetic analysis with two mitochondrial (COI and ND4 genes), a lineage that branched in *Gigantidas* in our multigenic phylogeny. Last, *B. elongatus* was maintained in *Bathymodiolus* because it falls close to *B. brevior* in the ITS2 data set of Olu-Le Roy et al. (2007). This species might, however, also be conspecific with *B. brevior* considering the very small genetic distance with species for this genetic marker.

Last, for four species no molecular data were available. First, no data are available for *I. argenteus,* but as it is the type species of the genus, it is de facto in this genus. Second, *Benthomodiolus abyssicola* was maintained in this genus because contrary to other lineages the shells appeared to be discriminant at the genus level (R. von Cosel, pers. inform.). Indeed, a new taxon added in this study was successfully attributed to this genus using the shell morphology, and the morphological data discussed in Lorion et al. (2010) also suggest that this lineage is morphologically diagnosable. Last, von Cosel and Janssen (2008)described two new species as belonging to the “childressi” group that we also tentatively maintained in *Gigantidas*.

### Character evolution

**Figure 3:**
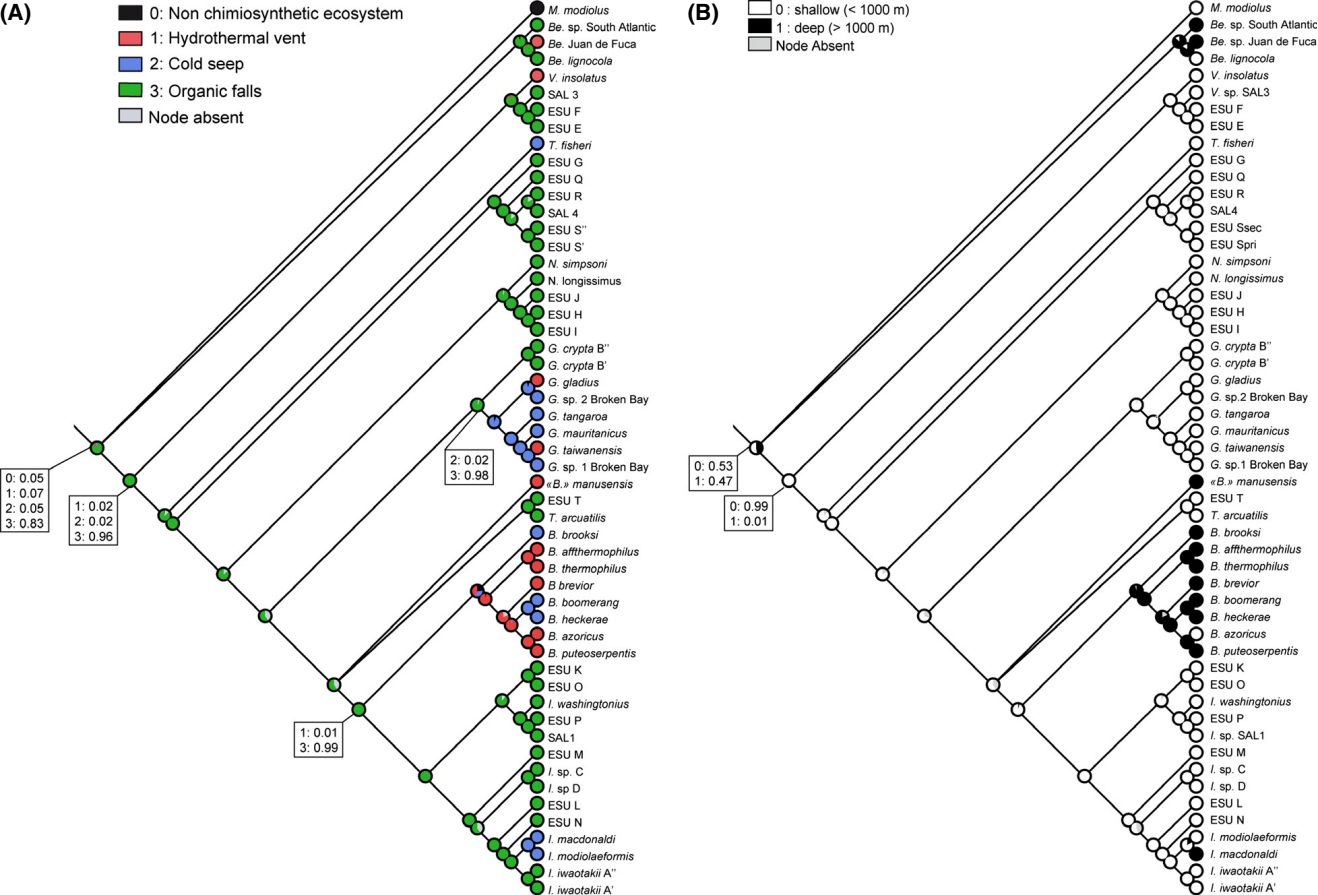
Bayesian cladogram of the combined data set with maximum likelihood estimates of ancestral character state for (A) habitat and (B) depth. Pie charts correspond to average likelihoods of each state. Numeric values of some nodes of interest are given.

The analysis confirmed that organic remains were the ancestral habitat for Bathymodiolinae (Fig. 3A). Furthermore, the colonization from organic falls to hydrothermal vents and/or cold seeps occurred at least in four lineages: *Benthomodiolus* (*Be*. sp. Juan de Fuca and *Be. lignocola* vs. *Be*. sp. South Atlantic), *Vulcanidas* (*V. insolatus* vs. the three unidentified species from sunken wood), *Gigantidas* (the *“childressi”* group, *G. gladius,* and *G*. sp Broken Bay vs. the *G. crypta* species complex), and at the divergence between *Bathymodiolus* and *Idas*. We could not conclude if the ancestral habitat was shallow or deep for bathymodiolin mussels (ancestral state: 0 = 0.53 and 1 = 0.47; Fig. 3B).

## Discussion

Including more taxa and more genes, we significantly improved the resolution of phylogenetic relationships among deep-sea mussels, both for many of the deeper nodes (among lineages) and for some of the internal nodes (within lineages). Most of the genera, as currently defined, are polyphyletic and/or paraphyletic and thus the current classification does not reflect evolutionary relationships. The existence of several lineages within Bathymodiolinae from seeps and vents was earlier hypothesized based on anatomical characters. For example, in the original description of *G. childerssi* (Gustafson et al. 1998), the unique combination of morphological characters was underlined but a new genus was not erected because the genetic distances were appraised too low to erect a new genus. Similarly, von Cosel and Olu (1998) doubted that the newly described species *B. boomerang* belong to the same lineage than *B. thermophilus* and suggested that part of the shared features (such as a straight intestine) were independently acquired. The first robust molecular studies (Jones and Vrijenhoek 2006) showed that *G. childressi* indeed belong to a distinct lineage, but that *B. boomerang* belongs to the “thermophilus” lineage. Although the classification used to date, although debated (von Cosel 2002; Génio et al. 2012), does not reflect evolutionary relationships and this has hindered comparative biology studies of deep-sea mussels. For example, Schultz et al. (2011) failed to efficiently cross-amplify microsatellite loci developed for “*B.” manusensis* in another species placed in the same genus (*B. heckerae*). We here show that these two species belong to two distant lineages and should be now classified in two distinct genera. The low cross-amplification success is thus explained by the fact that microsatellites only poorly cross-amplify among distant species and should be expected to be successfully transferred only to closely related species (Sharma et al. 2007).

Revising the taxonomy at the genus level, based on robust phylogenetic results, aims at guiding such comparative studies, and to challenge a priori assumptions about the likelihood of “evolutionary steps.” For example, the fact that embryos of the shallow water mussel *Mytilus edulis* can develop within a range of temperature and pressure encountered at vents is used by Mestre et al. (2009) as an argument to reject the “wooden-step” hypothesis. Mestre et al. (2009) assumed that an evolutionary step from eurythermal environments to cold stenothermal environments *is generally assumed highly unlikely*. However, the phylogenetic tree clearly shows that the evolutionary history of mussels from cold stenothermal chemosynthetic environments (i.e., both organic remains and cold seeps) and from the eurythermal environment found around hot deep-sea vents is tightly entangled. Moreover, our phylogenetic reconstruction shows that such evolutionary steps occurred more than once and are thus not so unlikely.

Based on this new phylogeny, organic remains are robustly supported as the ancestral habitat of Bathymodiolinae mussels (Lorion et al. 2010). Indeed, most of internal nodes of each genus are robustly assigned to the habitat “organic falls.” Only the ancestor of the *Bathymodiolus* clade is not statistically resolved. Moreover, whereas most habitat shifts occurred from bone and wood falls to vent and seep sites and a few occurred from seeps to vents, only one transition is inferred from vent to seep (ancestor of the sister-species *G. taiwanensis*/*G*. sp.1 Broken Bay). This result supports the “wooden steps” hypothesis proposed by Distel et al. (2000), in which the ancestors of seep and vent mussels were associated with organic falls. However, our analysis provided evidences that recurrent colonization events from organic remains to the harsh deep-sea seep and vent habitats occurred during the evolution of the Bathymodiolinae: a “multiple wooden steps” hypothesis would thus be more adequate. If some Bathymodiolinae species are able to inhabit both seeps and vents (e.g., *G. platifrons* and *G. japonicus* [Miyazaki et al. 2010; ]) or both seeps and sunken wood (Thubaut 2012), there is no species known to be able to colonize both organic remains and hydrothermal vents. Cold seeps seem to be an intermediate habitat for the colonization of deep vents, and a switch back from harsh vent habitats to organic falls is never inferred with sampling to date.

This phylogeny also allows a reevaluation of the evolutionary significance of some striking characters of the vent and seep mussels. For example, the repeated evolutionary shifts to vents and seeps are always associated with increase in mussels' size. The large size seems to be correlated with the habitat and thus could be a convergent evolution in independent lineages. Experimental results also suggest an important role of environmental parameters in the growth of Bathymodiolinae. For example, the size, the body condition, and the growth rate of *G. childressi* is correlated with the concentration of methane (Nix et al. 1995). These results were later confirmed by transplant experiments (Bergquist et al. 2004) in which mussels acquired nearly the characteristics of their host population. However, within the genus *Idas*, all the species either from organic falls or seeps and vents are very small (smaller than 1.5 cm c.f. [Lorion et al. 2010]). The polymorphism of some of the shells described from some species associated with organic falls (i.e., *V*. ESU E, *L*. ESU S, or *N. longissimus*) suggests that growth rate may also vary with environmental parameters. Additional experimental results are, however, needed to determine to what extent the size and/or growth rate of the small mussels from organic remains, belonging to each evolutionary lineage, may vary with the concentration of reduced compounds (such as hydrogen sulfide). The correlation of gigantism of mussels with seep and vent environments may, at least in part, explain why most of them have been placed in the genus *Bathymodiolus* and have received more attention from marine scientists than the small mussels from organic remains.

The inclusion of more data from various depths and environments shows that the evolution from shallow to deep (Craddock et al. 1995; Jones et al. 2006) is no more supported than the opposite hypothesis and that more samples from the deepest environments are needed to determine whether there is an evolutionary trend linked to the depth ranges. Data for organic falls from abyssal environments remain very scarce due to the difficulty of localizing them at the deep-sea floor. The only mean to obtain such data is to deploy experimental organic remains in abyssal environments (Voight and Segonzac 2012).

The phylogenetic interminglement of mussels from organic remains with mussels from seeps and vents challenges the scenarios drawn from previous phylogenetic analyses that were mainly based on a small and habitat-biased taxon sampling. For example, Fujiwara et al. (2010) and Miyazaki et al. (2010) suggest an evolutionary scenario in which seep and vent mussels were considered as a monophyletic lineage characterized by chemosynthetic endosymbionts. Based on the basal position of the few taxa associated with organic remains that have ectosymbionts, Fujiwara et al. (2010) suggested that extracellular symbiosis is an “earlier stage” in the evolution of Bathymodiolin than intracellular symbiosis. Although only little data are available on the position of the symbionts in the gill tissues for most of the species (see Table 2) (preventing any robust reconstruction of the ancestral states), our phylogeny shows that (1) several lineages include both types of symbionts and that (2) the symbionts position is not always related to habitats. For example, in the genus *Vulcanidas*, *V. insolatus* is suggested to harbor endosymbiotic bacteria (von Cosel and Marshall 2010), whereas ectosymbiotic bacteria were observed in the gill tissues of *Vulcanidas* sp. E sampled on sunken wood (Duperron et al. 2008b). Conversely, within *Gigantidas*, all species, either from vent and seeps or organic remains, have endosymbionts (Table 2). Finally, the two sister genera *Bathymodiolus* and *Idas*, as redefined here, display contrasted pattern of symbionts positions. Indeed, all *Bathymodiolus* species, from hot vents or cold seeps, have endosymbionts (reviewed in Duperron et al. 2009), whereas *Idas* species, mainly associated with organic remains – but also some from vents or seeps – have either endosymbionts (Baco-Taylor 2002; Baco and Smith 2003; Won et al. 2008) or ectosymbionts (Duperron et al. 2009). Although data are lacking for many species, our phylogenetic results suggest that endosymbiosis has been acquired more than once. In addition, the endosymbiotic bacteria observed in the gills of *G. crypta*, a species sampled either on sunken wood or bone remains, show that endosymbiosis should not be considered strictly as a specialization to vent or seep habitats.

Hydrothermal vents have been thoroughly explored in the past few decades and only a few new species are still being found, mainly from poorly explored biogeographic areas. The most recently described mussel species from vents that putatively fall into *Bathymodiolus* is *B. marisindicus* from Indian Ocean, an area that remains a yet underexplored biogeographic province even for vent habitats. However, molecular data suggested that *B. brevior* and *B. marisindicus* (Van Dover et al. 2001), but also *B. elongatus* (Olu-Le Roy et al. 2007), may be conspecific. The other recently described vent species fall putatively in *Gigantidas* (Hashimoto and Yamane 2005), *“Bathymodiolus”* (e.g., *“B.”. manusensis*), or in the new genus *Vulcanidas* (von Cosel and Marshall 2010). However, in these genera a lot more new species from seeps have been recently described (von Cosel and Janssen 2008) or have yet to be formally described. Similarly, the recent interest for organic falls added a bulk of species that are mostly not named. The sampling of deep-sea mussels from organic remains is far from being saturated (Lorion and Samadi 2010). Indeed, organic falls are still very poorly explored and we hypothesize that much more new species will be discovered from organic remains, but also from hydrocarbon seeps, than from vents.

The genus *Idas* is not only speciose but also ecologically diverse. It includes *I. modiolaeformis*, the species harboring the highest diversity of symbionts (Duperron et al. 2008a). Within this genus, species display either extra- or intracellular symbionts (Deming et al. 1997; Won et al. 2008; Duperron et al. 2009). The genus is found over wide geographic (Pacific, Atlantic, and Mediterranean) and bathymetric (180–2307 m) ranges and present in all kind of habitats. Generally, the population densities are low, although it may be locally abundant. For the relatively well-sampled species *I. iwaotakii*, the depth range within species covers more than 2000 m and the geographic range spreads over the Western Pacific. In contrast, the genus *Bathymodiolus,* sister lineage of *Idas,* includes species recovered in dense populations mainly from vents and a few from seeps. All species harbor intracellular symbionts and are restricted to depths greater than 800 m, with most of the species apparently restricted to a limited depth range (Fig. 1 and Table 2). Similarly, the *“Bathymodiolus”* lineage includes only two species (“*B.” manusensis* and “*B.” aduloides*). This lineage appears ecologically restricted: specimens were collected solely at vents or seeps, and only at sites below 1300 m (Iwasaki et al. 2006; Fujita et al. 2009; Kyuno et al. 2009; Miyazaki et al. 2010). In contrast, *Gigantidas* is well diversified, with a large number of species awaiting a formal description, and covers all kind of habitats (wood, bone, seep, and vent) over a quite large depth range (200–2222 m). Finally, *Benthomodiolus*, the sister genus of all other Bathymodiolinae, is still only known by a few species sampled mainly at very deep sites, both in the Atlantic and in the Pacific and both associated with organic falls and vents. Although no molecular data are available, at least another species (*Benthomodiolus abyssicola*) is mentioned in the Gulf of Panama at 3197 meters attached to wood (Wolff 1979). Wood falls below 1500 m have only been poorly explored (Samadi et al. 2010) and we can guess that exploration of deeper sites will bring more species in this lineage.

Overall, Bathymodiolinae lineages in which mussels are mainly associated with organic remains are more diversified and cover a larger range of ecological conditions, whereas lineages that mainly include vents species are less diverse and ecologically more restricted, but with very dense populations. This trend should be reinforced in the future as organic falls, particularly at depths below 1500 m, are still very poorly explored. Except for the *Bathymodiolus* lineage, the inferred ancestral habitat for the lineages was never the vent habitat. Vent species are ecologically successful, but, because of their low species richness and the apparent difficulty to switch back to other habitats, their evolutionary fate may be compromised. As a consequence, taking into account the species diversity associated with organic falls, we suggest that for mussels, adaptation to vents, although ecologically very efficient, is likely an evolutionary dead end.
